# The revised Bethesda guidelines: extent of utilization in a university hospital medical center with a cancer genetics program

**DOI:** 10.1186/1897-4287-8-9

**Published:** 2010-11-22

**Authors:** Aparna Mukherjee, Thomas J McGarrity, Francesca Ruggiero, Walter Koltun, Kevin McKenna, Lisa Poritz, Maria J Baker

**Affiliations:** 1 Department of Medicine, Division of Gastroenterology and Hepatology, Penn State Milton S. Hershey Medical Center, Hershey, USA; 2 Department of Pathology, Penn State Milton S. Hershey Medical Center, Hershey, USA; 3 Department of Surgery, Penn State Milton S. Hershey Medical Center, Hershey, USA; 4 Department of Medicine, Penn State Hershey Cancer Institute, Penn State Milton S. Hershey Medical Center, Hershey, USA

## Abstract

**Background:**

In 1996, the National Cancer Institute hosted an international workshop to develop criteria to identify patients with colorectal cancer who should be offered microsatellite instability (MSI) testing due to an increased risk for Hereditary Nonpolyposis Colorectal Cancer (HNPCC). These criteria were further modified in 2004 and became known as the revised Bethesda Guidelines. Our study aimed to retrospectively evaluate the percentage of patients diagnosed with HNPCC tumors in 2004 who met revised Bethesda criteria for MSI testing, who were referred for genetic counseling within our institution.

**Methods:**

All HNPCC tumors diagnosed in 2004 were identified by accessing CoPath, an internal database. Both the Tumor Registry and patients' electronic medical records were accessed to collect all relevant family history information. The list of patients who met at least one of the revised Bethesda criteria, who were candidates for MSI testing, was then cross-referenced with the database of patients referred for genetic counseling within our institution.

**Results:**

A total of 380 HNPCC-associated tumors were diagnosed at our institution during 2004 of which 41 (10.7%) met at least one of the revised Bethesda criteria. Eight (19.5%) of these patients were referred for cancer genetic counseling of which 2 (25%) were seen by a genetics professional. Ultimately, only 4.9% of patients eligible for MSI testing in 2004 were seen for genetic counseling.

**Conclusion:**

This retrospective study identified a number of barriers, both internal and external, which hindered the identification of individuals with HNPCC, thus limiting the ability to appropriately manage these high risk families.

## Background

Hereditary Nonpolyposis Colorectal Cancer (HNPCC), including Lynch syndrome (LS), is the most common hereditary condition associated with colorectal cancer, accounting for approximately 1-3% of all colorectal cancers [1-4]. In addition to early onset colorectal cancer (CRC), with an estimated lifetime risk of 70% in LS patients with a median age at diagnosis of 45 years, other cancers integral to this syndrome include endometrial, stomach, small bowel, ovarian, hepatobiliary tract, upper uroepithelial tract, and brain cancer [2,5,6]. Muir-Torre syndrome is a variant of HNPCC characterized by several cutaneous features including sebaceous adenomas, sebaceous carcinomas, and multiple keratoacanthomas, in addition to visceral cancers [2].

LS follows an autosomal dominant mode of inheritance, due to germline mutations in several DNA mismatch repair (MMR) genes, predominantly *MLH1 *and *MSH2*, but also *MSH6 *and less frequently *PMS2 *[2,7]. MMR genes encode proteins involved in the identification and repair of DNA mismatch errors, which inevitably arise during DNA replication. Defects within this pathway lead to microsatellite instability (MSI), the hallmark of HNPCC and LS tumors [8-10]. More than 90% of hereditary colorectal cancers associated with *MSH2 *or *MLH1 *mutations and 10-15% of sporadic colorectal cancers demonstrate high microsatellite instability using a panel of 5 microsatellite markers (MSH-H) [1,4,10-12].

MSI-H histology refers to characteristic histologic features of CRC associated with defective DNA MMR and includes a cribiform/solid (medullary) architectural pattern, infiltrating lymphocytes intimately associated with tumor cells, a Crohn's-like lymphocytic reaction, and mucinous/signet ring differentiation [13,14]. These histologic features can thus further aid in the identification of patients with HNPCC-associated colorectal cancer.

Given the high lifetime cancer risks associated with HNPCC, optimal selection of individuals for genetic screening and testing is paramount. Clinical criteria to help identify families with LS were established in 1991 and revised in 1999 [6,15].

To clarify the role of genetics and to elucidate clinicopathologic criteria to determine which patients should undergo genetic testing, the National Cancer Institute hosted an international workshop in 1996 that outlined a set of recommendations known as the Bethesda guidelines to help identify colorectal tumors that should be tested for MSI [16]. In 2004, the guidelines were amended and are the most accurate clinical criteria tested to date for identifying patients at risk for HNPCC [10,13,16,17] (see appendix 1). It has been estimated that the Bethesda guidelines will potentially target 15-20% of the total colorectal cancer burden [18]. Multiple studies have subsequently evaluated the clinical performance of the revised Bethesda guidelines and have confirmed that these recommendations constitute an efficient, cost-effective, and accurate means to identify patients at risk for HNPCC [11,17,19].

MSI testing could serve as a useful screening tool to identify families who may harbor a cancer-predisposing mutation for colorectal cancer, as well as other types of cancer [17]. Identifying MSI-H tumors should prompt referral for predictive genetic testing and with subsequent discovery of a gene mutation in select individuals, should lead to intensive surveillance strategies with improved outcomes for families with HNPCC [20,21].

The ability to identify these patients by applying the revised Bethesda guidelines, followed by referral to a genetics professional for microsatellite instability testing and germline testing, if indicated, would presumably be facilitated most readily in an institution that houses a Cancer Genetics Program. In our quality assurance study, we therefore aimed to retrospectively evaluate, over the course of one year, the actual percentage of patients diagnosed and treated for HNPCC-associated tumors who met revised Bethesda criteria, making them eligible for MSI testing, who were then referred for genetic counseling services within our tertiary care institution. This retrospective study also aimed to identify any barriers which may hinder the identification of individuals with HNPCC.

## Methods

The inclusion criteria for the study were adults, 18 years of age or older, who were diagnosed at our institution in 2004 with HNPCC-associated tumors including colorectal, endometrial, stomach, small intestine, ovarian, pancreas, ureter and renal pelvis, biliary tract, brain (glioblastoma) tumors, sebaceous gland adenomas/carcinomas and keratoacanthomas. Patients who were under 18 years of age or who had a diagnosis of familial adenomatous polyposis (FAP) were excluded from the study. Pathology specimens including carcinoid tumors, stromal tumors, islet cell tumors, primary peritoneal cancer, lymphoma, and brain tumors other than glioblastoma were also excluded since they are not typical malignancies characteristic of Lynch syndrome (Henry Lynch, personal communication).

All HNPCC-associated tumors diagnosed between January 1, 2004 and December 31, 2004 were identified by accessing CoPath, an internal database that classifies and tracks all anatomic pathology specimens and archives their gross and histologic findings. After identifying all eligible cases, both the Tumor Registry and each patient's electronic medical record were then accessed to gather all relevant family history information. The revised Bethesda criteria were applied to each patient case to determine which individuals fulfilled at least one of the criteria that would warrant MSI testing. The list of patients who met the revised Bethesda criteria, and were thus eligible for MSI testing, were then compared to those actually referred to the Cancer Genetics Program as detailed in the Patient Referral Database. Of those patients referred for cancer genetic services, we then determined which patients followed through with the genetics consult by accessing the Patient Appointment Database. For those patients who elected not to schedule an appointment for cancer genetic counseling, their reasoning, if provided, was documented in the Patient Referral Database.

Patient confidentiality was preserved and the study posed minimal risk to subjects. Full approval was obtained from the Human Subjects Protection Office prior to conducting this study.

## Results

As shown in Table 1, a total of 380 HNPCC-associated tumors were diagnosed at our institution between January 1 and December 31, 2004. Of the 380 tumor cases, 111 were colorectal cancers, 18 were glioblastomas, 7 were ureteral and renal pelvis tumors, 64 were uterine cancers, 36 were ovarian cancers, 21 were gastric tumors, 19 were small bowel carcinomas, 14 were biliary tract cancers, 62 were pancreatic cancers, and 29 were sebaceous adenomas or keratoacanthomas.

**Table 1:** Application of revised Bethesda guidelines to HNPCC tumors (2004)

Cases	**Meet Criteria (Criteria #)**^**2**^	Do Not Meet Criteria	Unknown	Total
Colorectal	21 (1, 2, 5)	39	51	111

Brain	0	11	7	18

Renal/Ureter	1 (5)	5	1	7

Uterus	2 (5)	46	16	64

Ovary	6 (2, 4, 5)	23	7	36

Stomach	3 (2, 4)	10	8	21

Small Bowel	3 (2, 4, 5)	7	9	19

Biliary	0	6	8	14

Pancreas	4 (2, 5)	31	27	62

Keratoacanthoma/SA^1^	1 (2)	16	11	28

Total	41	194	145	380

^1 ^Sebaceous adenoma

^2 ^The number in parentheses represents the specific criteria met (1 through 5) for each tumor type when the revised Bethesda guidelines were applied.

A total of 41 out of 380 cases (10.7%) met at least one of the revised Bethesda criteria and consisted of 21 colorectal, 1 ureteral/renal pelvis, 2 uterine, 6 ovarian, 3 gastric, 3 small bowel, and 4 pancreatic cancers, along with 1 keratoacanthoma case. In Table 1, the number in parentheses represents the specific numbered criteria (1 through 5) met for each tumor type when the revised Bethesda guidelines were applied.

Eight out of 41 patients who met at least 1 of the revised Bethesda criteria were referred for cancer genetic counseling, representing 19.5%. Of the 8 patients referred, 2 (25%) were seen by a member of the Cancer Genetics Program. The remaining 6 patients, although referred, did not follow through with an appointment. In some cases, the patients did not pursue genetic counseling because their insurance company did not recognize the credentials of the board certified genetic counselor/medical geneticist, and thus would not cover the consultation fee. Other patients did not pursue genetic counseling because they 1) didn't appreciate the potential benefits of the information, 2) expressed ambivalence about learning the information that the test could potentially provide and/or 3) expressed concerns about the potential for insurance and/or employment discrimination. Ultimately, only 4.9% of eligible patients who met revised Bethesda criteria for MSI testing in 2004 were seen for genetic counseling within our institution.

There were a large number of patients, 145 in total or 38% of all HNPCC tumor cases diagnosed in 2004, for whom it was not possible to determine if their personal and family history met at least one of the revised Bethesda criteria. In 20 of these patients, all of whom were diagnosed with colorectal cancer between 50 and 59 years of age, it was not possible to determine whether they met criteria 3 of the revised Bethesda guidelines since MSI-high histology was not consistently reported by the pathologist(s). The remainder of the 'unknown' patients, 125 in total, had limited or unknown family histories despite accessing both the Tumor Registry and the patient's medical record, thus precluding our ability to apply the revised Bethesda criteria.

## Discussion

Our study highlights several barriers, in applying the revised Bethesda criteria to identify patients at risk for Lynch syndrome based on a diagnosis of an HNPCC tumor. These barriers include limited family histories captured by health care providers and inconsistent reporting of MSI-H histology for colorectal cancer specimens by pathologists. Patient compliance represents another barrier identified lack of insurance coverage for genetic counseling services.

The results of our study demonstrate that 38% of patients (145/380) have incomplete recording of their family history of cancer and/or poorly characterized histology, thus hindering the application of the revised Bethesda criteria to determine eligibility for MSI testing. For these 38% of patients, it remains unknown whether they would have met the revised Bethesda criteria, had more information been made available. While approximately 11% of all HNPCC-related tumors (41/380) diagnosed over the course of one year at our tertiary care hospital met revised Bethesda criteria, only 20% of these (8/41) were referred for cancer genetic counseling, despite an active Cancer Genetics Program within our institution. In some of these cases, the health care provider did discuss consideration of cancer genetic counseling, but for a variety of reasons, the patients declined. Furthermore, among those patients referred, a significant number did not follow through with the counseling due to potential concerns for insurance and employment discrimination, financial barriers created by insurance companies, and possibly, a lack of appreciation for the long term benefits of surveillance and primary prevention in families with a hereditary predisposition to cancer.

The first major barrier we encountered in conducting this study was the cursory documentation of family history data in the medical chart by practitioners. Age at cancer diagnosis was rarely documented for relatives, cancer histories for 2^nd ^and 3^rd ^degree relatives were not recorded, and cancer types were not always specified. We accessed our institutional Tumor Registry database, which collects and maintains family history data on all patients first diagnosed and/or treated for a malignancy. However, the data collected relies solely on family history information previously recorded in the patient chart. The Tumor Registry does not directly interview patients. Furthermore, the Commission on Cancer and the State Department of Health does not mandate recording specific details regarding family history. We found that detailed pedigree information for extended relatives was not available. As a result, the application of the revised Bethesda criteria depended mostly on the affected patient's past medical history information, as opposed to family history information.

The ability to identify patients and their families at risk for HNPCC is strongly dependent on the clinician's recognition, understanding and application of the revised Bethesda criteria. These clinical criteria require the compilation of a detailed and thorough family history, the simplest and potentially most essential diagnostic task in assessing patients. Not surprisingly, previous studies have shown that even when a family history was suggestive of HNPCC, the significance of the data was either not appreciated by the clinician or the potential relevance of multiple HNPCC-related cancers in the same individual was overlooked [22,23].

We also noted that the Tumor Registry does not keep track of patients with non-melanoma skin cancers or precancerous lesions. We identified a total of 28 keratoacanthoma and sebaceous adenoma patients from the pathology database. In all instances dermatologists did not record a family history that could be found in the medical records. We identified one patient with a keratoacanthoma who met criteria #2 of the revised Bethesda guidelines since she was diagnosed with a metachronous colorectal cancer. This history, however, was not recognized by the dermatologist and the patient was not referred for cancer genetic counseling.

The low referral rate for cancer genetic counseling may, therefore, be partly attributed to poor recognition by clinicians of patients at-risk for HNPCC. Unlike several of the polyposis syndromes, HNPCC lacks striking phenotypic characteristics that would raise suspicion. Clinicians, therefore, must rely heavily upon a well-elucidated and well-documented family history, coupled with knowledge regarding the natural history of HNPCC and its association with multiple extracolonic tumors [2]. In one cohort, endometrial cancer predated the diagnosis of CRC in over 50% of HNPCC female patients [24]. Moreover, in the United States several cases have been successfully litigated when physicians failed to identify a patient at risk for a hereditary cancer predisposition syndrome [25].

Some practitioners may not appreciate the potential benefits of genetic testing for patients with cancer and their families. From a clinical management perspective, a number of studies have highlighted the importance of identifying patients and families with HNPCC. For example, in one study of patients with HNPCC, Järvinen et al. [21] showed that colorectal cancer surveillance reduced the risk and improved the survival of colorectal cancer in comparison to the control group who declined surveillance. Schmeler et al. [20] showed that prophylactic hysterectomy with bilateral salpingo-oophorectomy was an effective strategy for preventing endometrial and ovarian cancer in women with HNPCC. In comparison, Lindor and colleagues [26] demonstrated that patients who fulfill Amsterdam-1 criteria but without mismatch repair deficiency, and their kindred, can receive less intensive cancer surveillance than recommended for HNPCC patients, because these families have a significantly lower risk of colorectal cancer and no appreciable risk of extracolonic cancers, similar to the general population.

The second major barrier illuminated by our study was the inconsistent reporting by pathologists of specific features that define MSI-H histology repair. As a result of these histologic features not routinely being documented, it was not possible to analyze 20 of 111 colorectal cancer specimens diagnosed from 50 to 59 years of age (criteria #3). Following our request to retrospectively analyze these features for these 20 cases, the pathologist confirmed that 2 demonstrated tumor-infiltrating lymphocytes and 2 exhibited Crohn's-like lymphocytic reaction (20%). Multiple studies have demonstrated an improved prognosis in both sporadic and HNPCC cancers that demonstrate MSI-H histology [2,27-33]. Further, mismatch repair status predicted the benefit of adjuvant fluorouracil chemotherapy in colorectal cancer [34,35].

Pathologists play a central role in identifying HNPCC patients' age <50 years and HNPCC histology and could then reflex to MSI testing. This was proposed by Gologan et al. [11] in their prospective. Selecting specifically for age and histologic features, they found that out of 75 CRC cases examined, 23% were identified as MSI-H tumors. Furthermore, although the positive predictive value of histology suggestive of MSI-H was low at 32%, a total of 80% of the MSI-H CRCs had at least one of the criteria suggestive of MSI-H status [11]. An electronic reminder system for pathologists increased MSH tumor and Lynch syndrome patient identification [36].

Lastly, among those patients ultimately referred to the Cancer Genetics Program, few followed through with a consultation. This represents the third major barrier for the identification of HNPCC individuals. Unfortunately, Medicare and other major insurers do not currently provide payment for the board-certified medical professionals who are specifically trained to provide genetic counseling about the risks, benefits and limitations of testing. The American College of Medical Genetics and the National Society of Genetic Counselors are actively pursuing appropriate recognition of non-physician genetics providers. So far, this has resulted in masters and doctoral-trained genetics providers being eligible to apply for a National Provider Identifier (NPI) through the Centers for Medicare and Medicaid in 2006 and the development of a CPT code (96040) for genetic counseling services, effective January 1, 2007. Patients may also have concerns regarding the potential for insurance and/or employment discrimination.

To circumvent the barrier identified by our study and others, a more universal approach to molecular testing for HNPCC has been advocated. To circumvent the lack of detailed family history to identify HNPCC patients, a model for MSI testing of selected CRC tumors was found to be efficacious and cost-effective, identifying 2.2 times more patients compared to current practice [37]. In the study by Schofield et al. [38], MSI was used as an initial LS screening test followed by immunohistochemistry of MMR genes from CRC patients less than 60 years of age. From a cohort of 1,344 patients, 3.6% had germline mutations. Hampel et al. [39] evaluated the feasibility of screening for Lynch syndrome for all colorectal cancers. Limiting tumor analysis to patients who fulfill the revised Bethesda criteria failed to identify 28% of cases with Lynch syndrome. Lastly, the Evaluation of Genomic Applications in Practice and Prevention (EGAPP) Working Group commissioned an evidence-based review through the Agency for Healthcare Research and Quality to better understand the utility of DNA testing strategies in reducing morbidity and mortality from Lynch syndrome. Following a review of 104 studies published through 2006, they found sufficient evidence to recommend genetic testing for Lynch syndrome to all individuals with newly diagnosed colorectal cancer [40]. This group concluded that such a strategy could lead to improvement in medical management and clinical outcomes not only for patients, but also their relatives with Lynch syndrome. The EGAPP Working Group recommendations should not be viewed as discounting the value of family history but instead, adding a new pathway to identify high risk families [41]. The cost-effectiveness of four genetic testing strategies to indentify Lynch syndrome patients was developed from the U.S. healthcare system prospective. Universal testing would detect nearly twice as many cases as targeting younger patients with cost-effectiveness comparable with other preventive services [42].

Potential pitfalls to universal testing include the inability to definitively diagnose LS patients in an ever-changing genotype and phenotype landscape. For example, a recent series of LS families with MSH2 deficient tumors without detectable MSH2 mutations were found to have germ-line mutations in an upstream gene encoding for Ep-CAM [43]. Would these individuals previously been counseled as familial CRC type X [26]? Expansion of the LS phenotype to include prostate and bladder cancer has been documented, which increases the number of persons to be tested[44,45]. The ability of patients and at-risk relatives to deal with the emotional and psychological burdens of the complexity and uncertainty of genetic testing is understudied [46]. Finally, the fragmented U.S. healthcare system does not provide a central repository to carefully delineate the cancer risk for specific gene mutations or alert carriers of mutations of unknown significance should future research alter today's understanding of cancer risk.

## Conclusions

Our study identified several major barriers to the application of the revised Bethesda guidelines within our institution. We propose several recommendations that would allow the adoption of an improved systems-based practice.

First, a detailed family history should be captured by the initial health care provider who encounters the patient and the data should be recorded in the patient's electronic medical record. Second, concerted efforts need to be made to educate all providers about HNPCC and the importance of the revised Bethesda guidelines in identifying at-risk families. Third, emphasis should be placed on the pivotal role that pathologists can play in identifying at-risk patients by their consistent reporting of MSI histology. A departmental/institutional policy could be adopted that would direct pathologists to apply the MSI-H histology to all colorectal cancer specimens with subsequent recommendations when MSI testing should be pursued. Lastly, measures should be taken on a national level to eliminate barriers experienced by patients referred for genetic counseling. Continued efforts must be made to address the fear of insurance and employment discrimination by educating patients and providers about the Genetic Information Non-discrimination Act (GINA).

Overcoming the barriers identified in our study will enhance the detection of patients with Lynch syndrome, via the more traditional approach of high risk individuals being referred by astute clinical providers for consideration of genetic counseling and testing. In contrast, the EGAPP Working Group recommendations support a public health approach in which seemingly low risk individuals are screened in order to identify those high risk individuals within the population. These two approaches should be viewed as complementary, rather than one followed to the exclusion of the other, in order to optimize the identification of patients and their families with Lynch syndrome.

## Abbreviations

*Abbreviations used in this paper*: MSI: microsatellite instability; HNPCC: Hereditary Nonpolyposis Colorectal cancer; CRC: colorectal cancer; MSI-H: high microsatellite instability; FAP: Familial adenomatous polyposis; EGAPP: Evaluation of Genomic Applications in Practice and Prevention.

## Competing interests

The authors declare that they have no competing interests.

## Authors' contributions

AP carried out the study designs, data analysis and interpretation, and manuscript preparation. TM participated in study design, data analysis, manuscript preparation with significant contributions to all versions of the paper. FR, WK, KM, and LP contributed to the intellectual content of the paper including data analysis and interpretation, study design, and critical revision and review of the paper. MB participated in study design, data analysis, interpretation assistance in writing the first draft and significant contributions to all subsequent versions of the paper. All authors have read and approved the final manuscript.

## Appendix 1 (Umar et al. [13])

### Revised Bethesda guidelines for testing colorectal tumors for MSI

Tumors from individuals should be tested for MSI in the following situations:

1. Colorectal cancer diagnosed in a patient who is less than 50 yr of age

2. Presence of synchronous, metachronous colorectal, or other HNPCC-associated tumors (colorectal, endometrial, stomach, ovarian, pancreas, ureter and renal pelvis, biliary tract, small bowel, brain, and sebaceous gland adenomas and keratoacanthomas), regardless of age

3. Colorectal cancer with the MSI-high histology (presence of tumor-infiltrating lymphocytes, Crohn's-like lymphocytic reaction, mucinous/signet ring differentiation, or medullary growth pattern) diagnosed in a patient who is less than 60 yr of age

4. Colorectal cancer diagnosed in one or more first-degree relatives with an HNPCC-related tumor, with one of the cancers being diagnosed under age 50 yr

5. Colorectal cancer diagnosed in two or more first- or second-degree relatives with HNPCC-related tumors, regardless of age
